# Real-Time Messenger RNA Dynamics in *Bacillus subtilis*

**DOI:** 10.3389/fmicb.2021.760857

**Published:** 2021-11-18

**Authors:** Laura Sattler, Peter L. Graumann

**Affiliations:** Centre for Synthetic Microbiology (SYNMIKRO) and Fachbereich Chemie, Philipps-Universität Marburg, Marburg, Germany

**Keywords:** single-molecule tracking, mRNA dynamics, *Bacillus subtilis*, translation, ribosome dynamics

## Abstract

Messenger RNA molecules have been localized to different positions in cells and have been followed by time-lapse microscopy. We have used MS2-mVenus–labeled mRNA and single-particle tracking to obtain information on the dynamics of single-mRNA molecules in real time. Using single-molecule tracking, we show that several mRNA molecules visualized via two MS2-binding sites and MS2-mVenus expressed in *Bacillus subtilis* cells show free diffusion through the entire cell and constrained motion predominantly close to the cell membrane and at the polar regions of the cells. Because constrained motion of mRNAs likely reflects molecules complexed with ribosomes, our data support the idea that translation occurs at sites surrounding the nucleoids. Squared displacement analyses show the existence of at least two distinct populations of molecules with different diffusion constants or possibly of three populations, for example, freely mobile mRNAs, mRNAs in transition complexes, or in complex with polysomes. Diffusion constants between differently sized mRNAs did not differ dramatically and were much lower than that of cytosolic proteins. These data agree with the large size of mRNA molecules and suggest that, within the viscous cytoplasm, size variations do not translate into mobility differences. However, at observed diffusion constants, mRNA molecules would be able to reach all positions within cells in a frame of seconds. We did not observe strong differences in the location of confined motion for mRNAs encoding mostly soluble or membrane proteins, indicating that there is no strong bias for localization of membrane protein-encoding transcripts for the cell membrane.

## Importance

In contrast to dynamics of proteins, little is known about real-time motion of mRNA molecules in bacteria. We have added MS2-binding sites to several mRNAs in *B. subtilis* cells and followed motion of MS2-mVenus at a single-molecule level. We find that mRNAs show dynamics similar to those of ribosomes, despite their polymeric nature, and similar to those found in eukaryotic cells. Our data suggest that while mRNAs are preferentially translated at subpolar regions in cells, where also 70S ribosomes are accumulated, they can move through cells in a time frame of a few seconds. Thus, in general, mRNAs appear to be mobile to reach any position in the cell in a short time frame; they are preferentially located at the cell poles and close to the cell membrane, facilitating rapid translation and insertion of membrane proteins.

## Introduction

In the past, it has been a common model that transcription and translation occur at the same space and time in bacteria (Miller et al., 1970), because of the fact that, in general, bacteria are non-compartmentalized cells, unlike eukaryotes. Over the years, with the advance of powerful light microscopes and fluorescence labeling techniques, a new awareness of the organization and the inner structure of bacteria has arisen. Even though bacteria lack internal membrane systems similar to those found in eukaryotes, they possess a high degree of three-dimensional organization. Model bacteria such as *Escherichia coli* and *Bacillus subtilis* show a compacted structure called the nucleoid, containing the chromosome, which occupies the central space of the cell, but is absent at the cell poles or in the middle of large cells prior to cell division (Fisher et al., 2013). The cytoplasm is crowded with different enzymes diffusing through the cell (Cayley et al., 1991), whereas the RNA degradation machinery of *E. coli* and *B. subtilis* can be mainly found at the cell membrane (Miczak et al., 1991; Khemici et al., 2008; Hamouche et al., 2020). Transcription and translation occur spatially and temporarily coupled in *Caulobacter crescentus* cells (Campos and Jacobs-Wagner, 2013), in which the chromosome fills the entire cell, whereas in *E. coli* and *B. subtilis*, both processes occur largely separated from each other (Lewis et al., 2000), as transcription takes place mainly at the periphery of the nucleoid (Stracy et al., 2015), and translation at the cell poles. Different studies showed that only a minority of ribosomes, approximately 20%, are localized within the nucleoid, whereas 80% are localized in the cytoplasm surrounding the nucleoid, the membrane, and the poles (Mascarenhas et al., 2001; Chai et al., 2014). At the same time, only 4% of the RNA polymerase (RNAP) molecules and the ribosomes overlap in the nucleoid (Bakshi et al., 2012). Thus, in different bacterial species, transcription and translation can take place in close spatial proximity; that is, genes and ribosomes can be found at the same place, or largely separated, when the chromosome is organized as a nucleoid. In the latter case, the question arises how RNA moves from its places of synthesis on the nucleoids to the cell poles, and further, if mRNA might be translated near sites where the encoded protein is used, for example, in case of cell division proteins, or membrane proteins that specifically localize to the cell poles. Different models for mRNA localization have been discussed. For *C. crescentus*, the Jacobs–Wagner group has shown that mRNAs stay near their transcription sites, whose subcellular location in turn depends on its position of the chromosome, whose ordered arrangement serves as a spatial template (Montero Llopis et al., 2010). Considering a bacterium having a nucleoid, a gene locus would move out of the bulky nucleoid to the periphery, where RNAPs would transcribe the gene(s), and a coupling with translation (e.g., attenuation) would be possible (Stracy et al., 2015). A second principle model, suggested by the Amster–Choder group, is that the mRNA is translated near the localization, where its encoded protein would later on localize. They could identify three different patterns for RNA localization, along the membrane, near the poles and a helical distribution in the cytoplasm, for different proteins. Note that not all of the tested mRNAs could be found at the same spot as their corresponding protein product (Nevo-Dinur et al., 2011). Midcell localization was observed in another study, where an involvement of the signal-recognition pathway (SRP) for membrane proteins was assumed (dos Santos et al., 2012). In addition, mRNA encoding for membrane proteins has been found to be more associated with cell membranes than mRNA for soluble proteins (Benhalevy et al., 2015; Moffitt et al., 2016). Of note, different models could be possible in different bacteria species.

To address the question, where translation occurs in the Gram-positive bacterium *B. subtilis*, and to gain insight into the mode of diffusion of mRNAs, we decided to use the MS2 system of the bacteriophage (Peabody, 1993; Stockley et al., 1995). MS2 coat protein binds as a dimer to a 19-nt-long RNA loop structure, such that MS2 fused to fluorophore would bind to and visualize mRNA in living cells. In order to overcome low signal intensity, a large number of repeats of the binding site have been used, fixed cells or other larger arrays such as λN22 in eukaryotes (Schonberger et al., 2012). More recently, six repeats of the MS2-binding site have been commonly used (Kannaiah and Amster-Choder, 2016), which still considerably enlarge the molecules, also keeping in mind the many MS2-GFP molecules binding to the array. Because of inhibition or delayed degradation of mRNA (Garcia and Parker, 2015), different modifications were done over time by different groups (Tutucci et al., 2018). Other labeling techniques can be used with possibly less influence, such as fluorescence *in situ* hybridization (FISH) in fixed cells (Kannaiah and Amster-Choder, 2016), but do not allow to capture dynamics. In order to limit adverse effects, we decided to use only one or two MS2-binding sites fused to the 3′ end of the mRNA sequence. Our goal was to determine if motion of mRNA can be explained by assuming Brownian motion, using *B. subtilis* as a model bacterium, or if the polymers move largely by anomalous diffusion, or show no motion at all. We also set out to attempt determining diffusion constants, in order to find out if large mRNAs show markedly different dynamics than small molecules. We also wished to address the question of mRNA operons encoding for membrane proteins membrane—proximal localization can be observed, as was described for some cases before, or if mRNAs can be mobile throughout the non-compartmentalized bacterial cells. Using single-molecule tracking (SMT), we found evidence for different populations having distinct average diffusion constants, indicating low mobility of mRNA bound to a ribosome during translation and mobile populations, in which mRNA could be bound to a partially assembled ribosome or even freely diffusing molecules. Our data show that, for model mRNAs, diffusion can occur throughout the entire cells, whereas static motion occurs mostly at the periphery of the cell. Our data are in agreement with translation occurring at specific subcellular places, but show a high degree of mobility to reach these sites.

## Results

### Use of MS2-mVenus to Investigate mRNA Diffusing at Single-Molecule Level in Live Cells

In contrast to other studies using the MS2 system for mRNA detection, in this work, we used only one or two MS2-binding sites instead of six or more (Nevo-Dinur et al., 2011; van Gijtenbeek et al., 2016) and MS2 coat protein (Peabody, 1993; Stockley et al., 1995) fused to the bright, monomeric GFP derivate mVenus (Kremers et al., 2006). Rather than using fixed cells (Femino et al., 1998) and bulky MS2-GFP tags or slow acquisition speeds (4 Hz) (Golding and Cox, 2004), we imaged using slim field illumination and stream acquisition in the milliseconds range (Rosch et al., 2018; Hernandez-Tamayo et al., 2019; Burghard-Schrod et al., 2020). We reasoned that at an integration time of 75 ms, we would not track freely diffusing MS2-mVenus molecules, for which ≤ 20 ms would be required [5 ms is necessary to track free mVenus, (Schibany et al., 2018)], but predominantly track MS2 molecules bound to their target mRNA molecules. To test this assumption, we imaged MS2-mVenus via SMT with two different exposure times. We chose 8 ms to capture freely diffusive molecules, or 75 ms, where we would likely miss out freely diffusive molecules.

**FIGURE 1:**
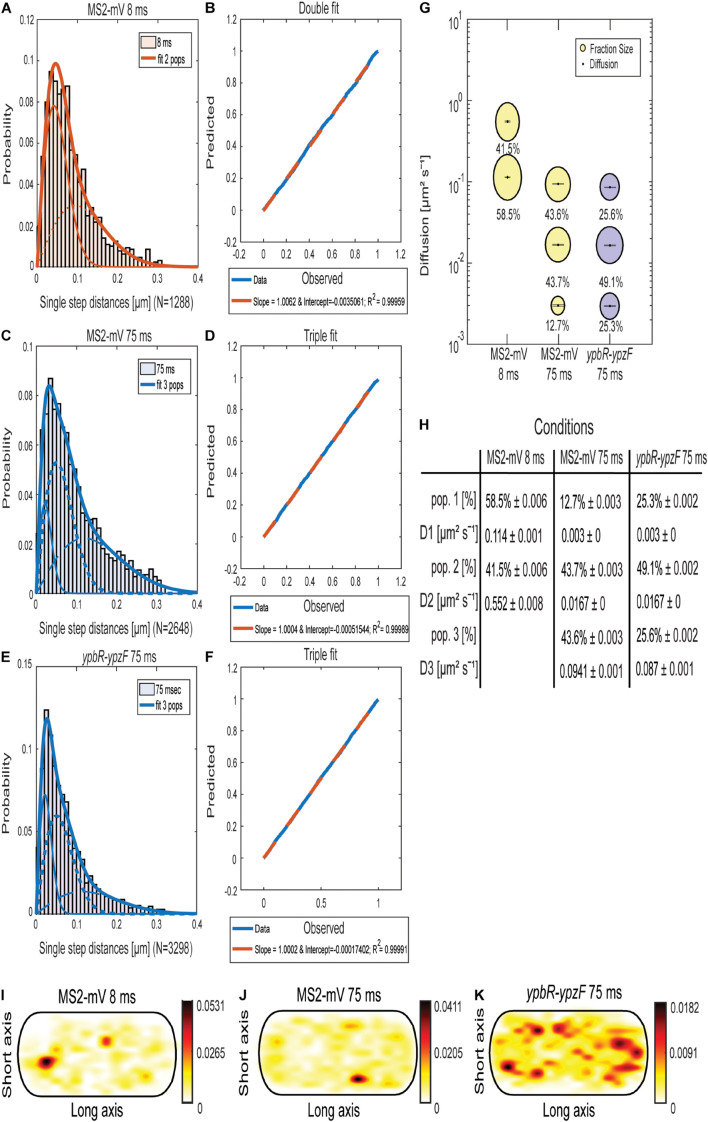
Analyses of single-molecule dynamics of MS2-mVenus expressed in *B. subtilis* cells during exponential growth. **(A,C,E)** Jump distance analysis shows probability of displacements 8-ms acquisition **(A)** or 75-ms acquisition **(C,E)**. **(B,D,F)** Probability–probability plot displays the goodness of fit of predicted (red dotted lines) and determined data (blue solid lines). Triple Rayleigh fit models are shown in blue; double fits are depicted in red. Different dotted lines represent the subpopulations; solid lines represent the sum of the subpopulations. **(G)**. Bubble plot shows the size of the fractions (proportional to the area) and corresponding diffusion coefficients. MS2-mVenus only is shown in yellow, in the presence of *ypbR-ypzF* carrying two binding sites in purple. **(H)** Table showing population size (pop.) and diffusion constants “D” of MS2-mV only at two different acquisition times, and of specifically tagged *ypbR-ypzF* mRNA. **(I–K)** Heat maps for MS2-mVenus tracked with 8 ms **(I)**, 75 ms **(J)**, and in the presence of tagged *ypbR-ypzF* mRNA. White to red: low to high probability of distribution of tracks.

Jump distance (JD) analysis (Figures 1A,C,E) is based on squared displacement analyses (Weimann et al., 2013; Rösch et al., 2018), showing the probability of particles taking certain steps during a given time interval, assuming two-dimensional Brownian motion. We used several tests in order to evaluate if a single or more populations with an average diffusion constant can explain the observed JD distribution, using Rayleigh distributions. Probability–probability plots shown in Figures 1B,D,F plot the deviation between observed data (blue line) and modeled data (red dotted line), and lack of deviation indicates a high quality for the goodness of fit (Rösch et al., 2018). Based on tests and the probability plots, MS2-mVenus tracked with 8-ms exposure time showed two populations, which best explain the data (Figures 1A,B), having diffusion coefficients (DCs) of D_2_ with 0.55 μm^2^ s^–1^, likely freely diffusive MS2-mVenus, and D_1_ of 0.11 μm^2^ s^–1^, that is, a slow-mobile population, likely consisting of MS2 bound to mRNA (Figures 1G,H). A DC of 0.55 μm^2^ s^–1^ is surprisingly low for a small protein fused to mVenus, but at the acquisition speed used, we do not believe that our analyses are hampered by technical limitations. Supplementary Figure 1 shows that assuming a third population does not increase the goodness of fit (compare panels B and D) and was therefore discarded.

**FIGURE 2:**
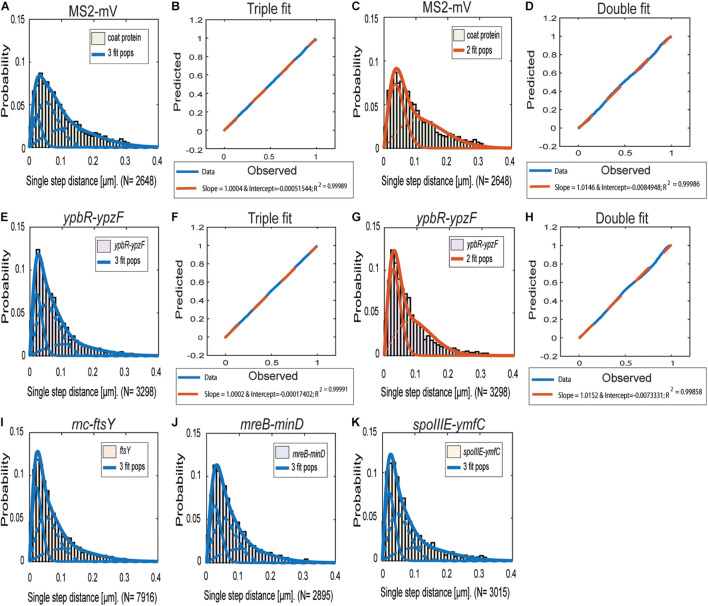
Jump distance analyses of four MS2-tagged mRNAs. Triple fits are shown in blue; double fits in red. **(A–D)**, Cells expressing MS2-mVenus only. **(E–K)** Cells additionally containing MS2-binding sites fused to mRNAs. **(E–H)** MS2-mVenus + *ypbR-ypzF* **(I)** MS2-mVenus + *ftsY*_MS2–binding site 2 ×, **(J)** MS2-mVenus + *mreB-minD*_MS2–binding site 2 ×, and **(K)** MS2-mVenus + *spoIIIE-ymfC*_MS2–binding site 2 ×.

On the other hand, for MS2-mVenus tracked with an exposure time of 75 ms, three populations can explain the data better than assuming two populations (Supplementary Figures 1E–H). The most mobile population of MS2-mVenus at 75 ms had a diffusion constant (D) of 0.094 μm^2^ s^–1^, very similar to that of the slower population tracked with 8 ms (Figure 1G). The intermediate population D_2_ had a *D* of 0.012 μm^2^ s^–1^ and a size of 43.7%, similar to the fast-mobile population with 43.6%. D_1_ with 0.003 μm^2^ s^–1^ weighed only 12.7%. Most likely, the latter population represents MS2 bound to mRNA being translated by polysomes, the largest structure expected for mRNA to be present in. Very similar DCs were obtained for cells expressing *ypbR-ypzF* mRNA having two MS2-binding sites (Figures 1E,F, 2E–H). Here, the triple fit covered best the whole data set. These data suggest that MS2 protein binds to mRNA other than its target mRNA with considerable affinity. Although this caveat compromises specific claims, we found that the static fraction is much larger for cells carrying mRNA with MS2-binding sites, 25% of MS2-mVenus molecules showed static motion compared to 13% in cells lacking the MS2-binding site, and likewise, the intermediate mobile population was larger (49% vs. 44%), whereas the high mobility fraction was smaller (26% vs. 44% in cells lacking MS2-binding sites). These findings suggest that the high mobility fraction represents mostly MS2 bound to non-specifically recognized targets, whereas the other two fractions contain a large portion of MS2 bound to its specifically recognized mRNAs.

As a further argument for a considerable degree of specificity, we analyzed the presence of MS2-mVenus molecules in an average size cell of 3 × 1 μm, into which all tracks obtained from hundreds of cells are projected. For example, a “heat map,” where white areas show a low probability of distribution and red to black a high concentration of molecules, shows that MS2-mVenus diffuses throughout the cell with no clear preference to a certain area (Figures 1I,J), whereas the presence of the MS2-binding sites on *ybpR-ypzF* mRNA shows a preferential localization toward the periphery of cells (Figure 1K). Of note, expression of MS2-mVenus at low level does not strongly affect exponential growth, but cells reached a lower density than cells lacking the fusion protein (Supplementary Figure 2). We speculate that MS2 might bind to one ribosomal RNA, a highly abundant molecule that would strongly compete with few mRNAs carrying two MS2-binding sites. With this caveat in mind, we went on to analyze if differences might be observed for different mRNA molecules.

### mRNA Moves as Three Distinctly Measurable Fractions of Mobility Within Cells

We analyzed four different mRNAs, all of the operons. The *ypbR-ypzF* operon comprises open reading frames (ORFs) for soluble and membrane-associated proteins (Table 1). *ypbR* encodes for DynA, a soluble protein involved in membrane fusion after lesions (Sawant et al., 2016), and appears to aid in membrane fusion of the invaginating division septum (Dempwolff et al., 2012). The products of *ypbS* and *ypzF* are of presently unknown function. FtsY is the membrane-associated receptor for the signal recognition particle (Braig et al., 2009) and localizes at the membrane, but also diffuses through the cell (Mayer et al., 2021); RNase Y is also membrane-associated (Hamouche et al., 2020), whereas SMC localizes to the nucleoids (Schibany et al., 2018). The *mreB-minD* operon comprises genes for cell shape-determining proteins MreB (soluble), MreC, and MreD (membrane proteins), part of the Rod complex for lateral cell wall synthesis (Dominguez-Escobar et al., 2011; Dersch et al., 2020). MreB forms filamentous structures underneath the cell membrane (Reimold et al., 2013). MinC and MinD, encoded at the 3′ end of the operon, are part of the membrane-associated Min system (Yu et al., 2020) and function as cell division inhibitors (Strahl and Hamoen, 2010), localizing at the septum and cell poles. The fourth mRNA, operon *spoIIIE-ymfC*, encodes for YmfC, a transcription factor of the GntR family (Resch et al., 2010) and membrane protein SpoIIIE, an ATP-dependent dsDNA translocase (Fleming et al., 2010; El Najjar et al., 2018). MreB and MreC are essential proteins, and the deletion of FtsY leads to extremely slow growth of *B. subtilis*. Addition of two MS2-binding sites did not considerably slow down growth of cells compared with cells expressing only MS2-mVenus (Supplementary Figure 2), indicating that both mRNAs retain functionality. Although we cannot test for functionality of the other two tagged mRNAs during exponential growth (note that SpoIIIE is constitutively expressed and plays an additional role during sporulation), we assume that the two binding sites do not cause a major defect in terms of mRNA dynamics.

**TABLE 1:** List of mRNAs analyzed in this study and their encoded genes.

	**Length**	**Genes**	**Essential**	**Encodes for**
*comN-secDF* ^1^	2950 bp	*comN, secDF*	No	Membrane proteins
*rnc-ftsY* ^1,2^	5477 bp	*rnc, smc, ftsY*	Yes	Soluble and membrane-associated protein
*hag* ^1^	915 bp	*hag*	No	Extracellular, soluble protein
*mreB-minD* ^1,2^	3894 bp	*mreB, mreC, mreD, minC, minD*	Yes	Soluble and membrane proteins
*rplJ-rplL* ^1^	873 bp	*rplJ, rplL*	Yes	Soluble proteins
*rplK-rplA* ^1^	1264 bp	*rplK, rplA*	No, but severe phenotype	Soluble proteins
*spoIIIE-ymfC* ^2^	3191 bp	*spoIIIE, ymfC*	No	Soluble and membrane proteins
*ylxM-rplS* ^1^	4395 bp	*ylxM, ffh, rpsP, ylqC, ylgD, rimM, trmD, rplS*	Yes	Soluble proteins
*ypbR-ypzF* ^1,2^	4059 bp	*ypbR, ypbS, ypzF*	No	Soluble and membrane-associated proteins

*^1^Artificial mRNAs with one MS2-binding site and 1,700-bp plasmid sequence (not added in the table). ^2^mRNAs with two MS2-binding sites.*

Figures 2A–G reinforce the idea that the distribution of *ypbR-ypzF* tracks is best explained assuming the presence of three populations tagged mRNAs (Figures 2I–K). The same holds true for the other three tagged mRNAs, indicating that three distinct diffusion constants could be a general property for mRNA molecules.

**FIGURE 3:**
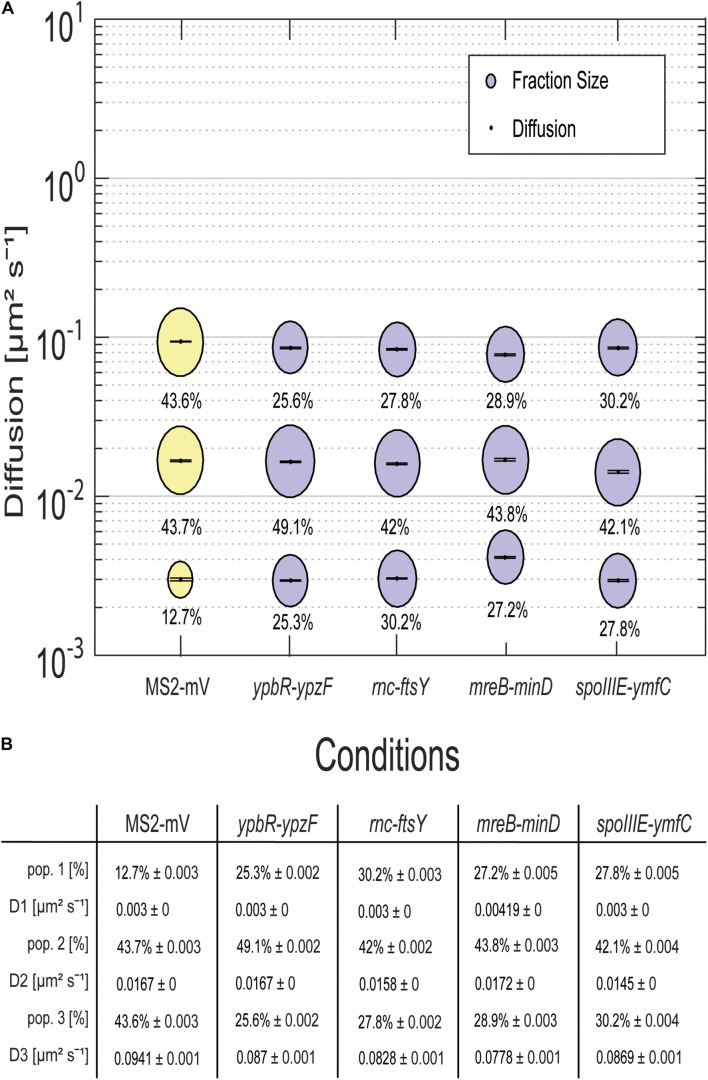
Dynamics of different mRNAs for soluble, membrane, and membrane-associated proteins in comparison to MS2-mVenus. For the determination of the diffusive coefficient and the fraction size, SQD was used. **(A)** The bubble plot shows the size of the fraction where each bubble is proportional to the area of its corresponding diffusion coefficients. MS2-mVenus alone is shown in yellow, whereas the different mRNAs are shown in purple. Table **(B)** states the SQD results.

For all tagged mRNAs, the diffusion constants for the most mobile populations D_3_ are similar between MS2-mVenus alone and bound to the mRNAs. In MS2-mVenus, the mobile fraction has *D* = 0.094 μm^2^ s^–1^, *ypbR-ypzF* 0.09 μm^2^ s^–1^, *rnc-ftsY* 0.083 μm^2^ s^–1^, *mreB-minD* 0.078 μm^2^ s^–1^, and *spoIIIE-ymfC* 0.09 μm^2^ s^–1^ (Figure 3). Note that errors stated in Figure 3B refer to fitting errors, whereas all data are combined from tracks of three independent biological replicates (for statistics, see Supplementary Table 1). The intermediate mobile diffusion constant (D_2_) is roughly one order of magnitude lower, with 0.019 μm^2^ s^–1^ for MS2-mVenus and *ypbR-ypzF.* For *rnc-ftsY, D* is 0.016 μm^2^ s^–1^ for *mreB-minD* 0.017 μm^2^ s^–1^, and for *spoIIIE-ymfC*, *D* = 0.015 μm^2^ s^–1^ (Figure 3). Those two populations diffuse faster or slower compared to a study about mRNA diffusion, suggesting 0.05 μm^2^ s^–1^ for mRNAs moving out of the nucleoid into the ribosome-rich area in *E. coli* (Castellana et al., 2016). Thus, for *ypbR-ypzF, rnc-ftsY, mreB-minD*, and *spoIIIE-ypzF*, the high mobility population could represent free mRNA, and the intermediate-mobile population could consist of partially assembled ribosomes, where the mRNA is already bound to the smaller subunit S30, before the large subunit starts to associate (Gualerzi and Pon, 2015). We will discuss SMT of the ribosomal protein L1 later on. The static population D_3_ does not differ considerably between MS2-mVenus expressed alone or with mRNA containing binding sites (Figure 3). The diffusive coefficient with 0.003 μm^2^ s^–1^ for MS2-mVenus, *ypbR-ypzF, rnc-ftsY*, and *spoIIIE-ymfC* and/or with 0.0042 μm^2^ s^–1^ for *mreB-minD* is extremely low, suggesting engagement of mRNA with polysomes. Of note, our measured diffusion constants are in good agreement with those found for eukaryotic cells (Katz et al., 2016; Yan et al., 2016).

MS2-mVenus alone has a large high-mobility population of 43.6%, and a large intermediate mobile fraction (43.7%), but a small static fraction (12.7%). For *ypbR-ypzF* compared with MS2-mVenus control, the highly mobile population decreases to 25.6% ± 0.002%, whereas the static population increases to 25.3% ± 0.002%. Also, the intermediate mobile population with 49.1% is larger than that for “MS2-mVenus-only.” Static populations of the other mRNAs were also more than twofold larger than that of MS2-mVenus alone (Figure 3), suggesting that static fractions are dominated by MS2-mVenus specifically bound to its cognate mRNA. Based on this, we can assume that approximately one-third to half of our data in the strains expressing tagged mRNA are tracked MS2 coat protein bound to the binding site on the chosen mRNA, and leaving a large but not overwhelming degree of background noise of MS2 coat protein binding to unspecific targets, most likely RNA, in the cells.

Considering the size of mRNAs, the two mRNAs operons*ypbR-ypzF* with 4,059 bp and *mreB-minD* with 3,894 bp, and larger mRNAs *rnc-ftsY* with 5,477 bp (or 4,627 bp whenthe *smc* promoter is used) and the smaller operon *spoIIIE-ymfC* with 3191 bp (Table 1), show similar diffusion constants (Figure 3). Thus, to a first approximation, the size of mRNA does not appear to play a decisive role for mRNA diffusion, or for the mobility of assembled polysomes.

### Single-Molecule Tracking Reveals a Similar Behavior of Ribosomal Protein L1 Compared to mRNA

**FIGURE 4:**
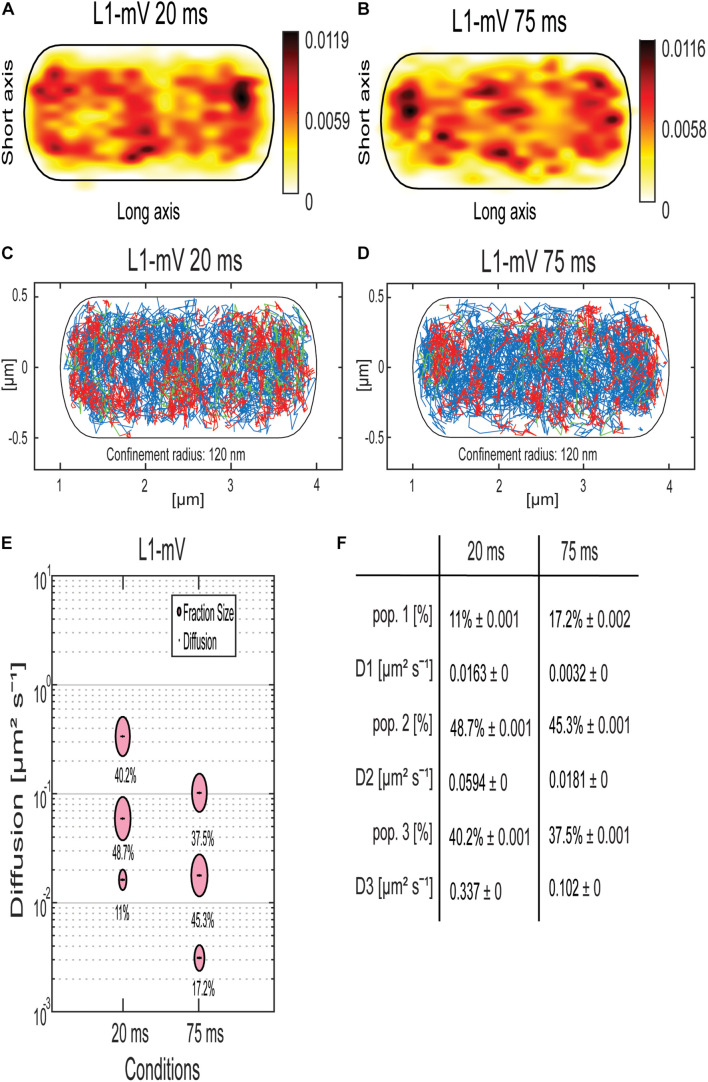
Analyses of a ribosomal L1-mVenus protein fusion using SMT with 20- and 75-ms stream acquisition. **(A,B)** all tracks of L1-mVenus are projected into a standardized cell of 1 × 3 μm. From white to red, low to high probability of localization for tracking with an exposure time of 20 ms **(A)** and 75 ms **(B)**. **(C,D)** Confinement maps. Blue represents freely diffusive tracks, red tracks showing confined motion within a 120-nm circle for a minimum of eight steps, and green tracks showing mixed behavior. **(E)** The bubble plot shows the size of the fractions; each bubble is proportional to the area of its corresponding diffusion coefficients. **(F)** Table showing population size and diffusion constants at different acquisition times. Squared displacement analysis was used for the determination of the diffusion constants and fraction sizes.

To address the nature of the different populations of MS2 tagged mRNA, we performed SMT of the ribosomal protein L1, which is part of the large subunit of the ribosome (Akanuma et al., 2012; Rosenberg et al., 2012). L1-BFP has been shown to be fully functional and to localize mainly at the cell poles and around the nucleoids using epifluorescence (Mascarenhas et al., 2001). We performed SMT using 75-ms stream acquisition for comparison with the mRNA data and 20 ms to be able to detect possibly freely diffusive L1. Highest density of L1-mVenus molecules was observed at the cell poles, septum area, and at the membrane (Figures 4A,B), in agreement with earlier epifluorescence experiments (Mascarenhas et al., 2001).

Jump distance (JD) analyses suggest that assuming two populations for L1-mVenus can explain the observed data well, but three populations increase the quality of the overall fit (Supplementary Figure 3). square displacement analysis (SQD) analysis shows that L1, tracked with 20-ms exposure time, has a high-mobile population of 40.2%, with *D* = 0.34 μm^2^ s^–1^, a slow mobile population of 48.7%, with *D* = 0.06 μm^2^ s^–1^, and a static population of only 11%, with *D* = 0.016 μm^2^ s^–1^ (Figures 4E,F). We can assume that the high-mobile fraction consists of L1 within diffusing large subunits, as D_3_ is similar to that determined by the Elf group for free subunits that diffuse through the entire cell (Sanamrad et al., 2014). The other two populations likely represent L1 bound to 70S subunits plus mRNA. In *E. coli*, a DC of 0.04 μm^2^ s^–1^ was assumed for a translating ribosome (Bakshi et al., 2012). Our investigation suggests that more than a single form of translating ribosomes exists or different forms of ribosome/mRNA complexes with distinct mobilities. In comparison, L1-mVenus tracked with longer integration time, DCs as all fractions decreased, likely because many fast-diffusing L1 molecules (e.g., in the free large subunit) are not captured at 75-ms integration time. The static population has a lower DC at 75 ms compared to 25 ms, with *D* = 0.003 μm^2^ s^–1^, because it can be more accurately determined at slower stream acquisition.

Interestingly, at 75-ms integration time, the DC of the static population of L1 with 0.003 μm^2^ s^–1^ and of the mRNAs with 0.003 μm^2^ s^–1^ for *ypbR-ypzF, rnc-ftsY*, and *spoIIIE-ymfC* and 0.004 μm^2^ s^–1^ for *mreB-minD* were very similar (Figure 4F). Thus, we propose that this population might be polysomes in full swing and/or mRNA for membrane proteins being translated at the SecYEG translocon after delivery through the SRP system.

### Analyses of Confined Motion Shows Preferential Low Mobility of mRNA at Sites Corresponding to Translating Ribosomes

**FIGURE 5:**
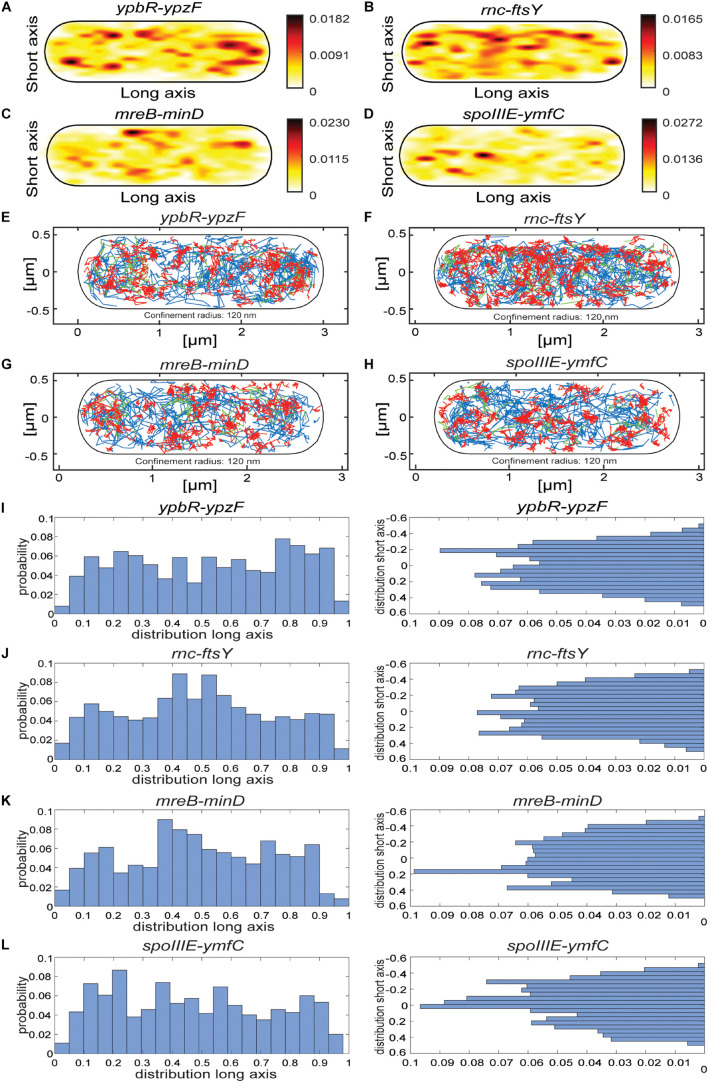
Localization patterns of different mRNAs tagged with two MS2-binding sites. All tracks of MS2-mVenus bound to 2 × MS2-binding site tagged mRNAs are projected into a standardized cell of 1 × 3 μm. For heat maps, white to red represent low to high probability of localization of molecules. **(A)** MS2-mVenus + *ypbR-ypzF*_MS2–binding site 2 ×, **(B)** MS2-mVenus + *ftsY*_MS2–binding site 2 ×, **(C)** MS2-mVenus + *mreB-minD*_MS2–binding site 2 ×, **(D)** MS2-mVenus + *spoIIIE-ymfC*_MS2–binding site 2 ×. For confinement maps, blue represents freely diffusive tracks, red tracks restricted to a confined motion within a 120-nm circle for a minimum of eight steps, and green tracks with mixed behavior between mobile and confined motion. **(E)** MS2-mVenus + *ypbR-ypzF*_MS2–binding site 2 ×, **(F)** MS2-mVenus + *ftsY*_MS2–binding site 2 ×, **(G)** MS2-mVenus + *mreB-minD*_MS2–binding site 2 ×, and **(H)** MS2-mVenus + *spoIIIE-ymfC*_MS2–binding site 2 ×. **(I–L)** Histograms showing the probability of confined tracks occurring along long (*x*) – or short (*y*) axis of cells. Histograms correspond to panels **(E–H)**.

Heat maps for all four analyzed mRNAs showed a tendency for a preferential localization toward the cell periphery (Figures 5A–D), different from the relatively even distribution of MS2-mVenus only (Figure 1J), with the *ypbR-ypzF* transcript revealing the strongest accumulation at the cell poles (Figure 5A). In order to gain spatial information of different dynamics within the cell, we set up confinement maps, which visualize low molecule mobility in cells. Confined motion was assumed for molecules that stay within in a circle of 120 nm (roughly three times the localization error in this study) for a minimum of eight steps and longer. Such tracks represent molecules that stay associated with a defined subcellular position, likely bound to a large, static complex and are represented by red tracks in Figures 5E–H. Of note, these molecules will largely overlap with the population of molecules showing the slowest diffusion constant and thus to 50% and more represent MS2-mVenus bound to its specific mRNA molecule rather than non-specifically bound MS2-mVenus (see above). While freely diffusing molecules, indicated by blue tracks, are found throughout the cells, confined motion is enriched at the cell periphery and is depleted from the central places of the cell (Figures 5E–H). The exception is the cell middle, whereas in large cells (tracks of all sizes of cells are projected into the standardized cell), the segregated nucleoids make space for the invaginating septum, and where ribosomes are also accumulated. The trend for depletion at spaces of nucleoids is also seen for L1 protein representing the ribosomes (Figures 4C,D). As confined motion likely represents translating polysomes, that of labeled mRNAs likewise will account for transcripts being actively translated.

According to the model of the Amster–Choder group (Nevo-Dinur et al., 2011), mRNAs may be enriched near the area where an encoded protein will be active. For the four mRNA molecules, we found small but noticeable differences in the probability of confined motion, as apparent from the histograms of the short (*y*) cell axis or long (*x*) axis distributions (Figures 5I–L). *ypbR-ypzF* mRNA showed a relatively even distribution with depletion of confined tracks from the cell center (Figure 5J). For *rnc-ftsY*, enrichment at the septal area can be seen in the *x*-axis histogram (Figure 5I). FtsY has been shown to diffuse through the entire cell in *Shewanella putrefaciens*, but to move in confined motion close to the cell membrane (Mayer et al., 2021). Thus, while enrichment at the septal area might suggest preferred translation at this site in large cells commencing division, freely diffusive FtsY can easily reach this place within few seconds. For *mreB-minD* and for *spoIIIE-ymfC*, we observed polar enrichment (Figures 5K,L), as judged from the *x*-axis distribution.

Mixed behavior between mobile and confined localization, indicated by green tracks in Figures 5E–H, is much more rarely observed than either confined or free mobility. This indicates that molecules only rarely switch between free diffusion and confinement, but rather stay in either state of mobility for an extended time.

### Rifampicin Stress Affects the Localization of *spoIIIE-ymfC* mRNA

**FIGURE 6:**
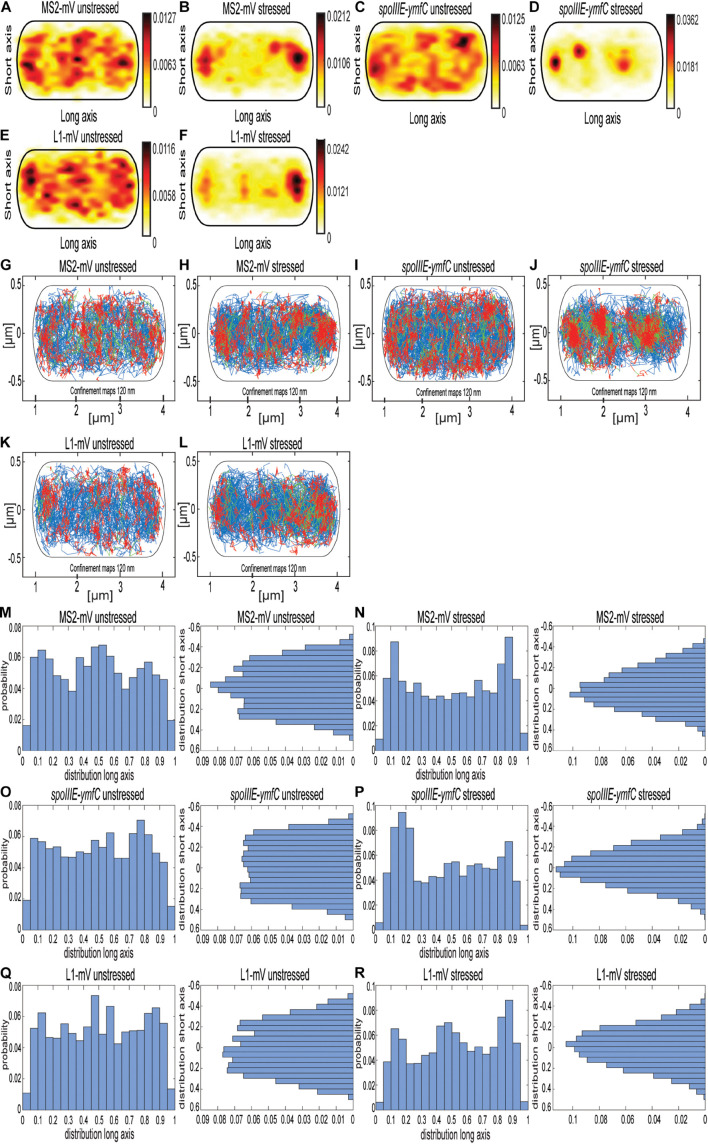
Localization of MS2-mVenus, *spoIIIE-ymfC*, and L1-mVenus with and without 50 mg rifampicin. All tracks of MS2-mVenus alone **(A,G)** or bound to 2 × MS2-binding site tagged mRNA *spoIIIE-ymfC* **(C,I)** or the ribosomal protein L1-mVenus **(E,K)** are projected into a standardized cell of 1 × 3 μm. **(B,H)** *B. subtilis* cells expressing MS2-mVenus alone stressed for 40 min with 50 μg/mL rifampicin **(D,J)** or bound to 2 × MS2-binding site tagged mRNA *spoIIIE-ymfC* or **(F,L)** ribosomal protein L1-mVenus. **(A–F)** Heat maps, from white to red, low to the probability of localization of molecules. **(G–L)** Confinement maps, blue represents freely diffusive tracks, red tracks restricted to confined motion within a circle of 120 nm for a minimum of eight steps, and green tracks having mixed behavior. **(M–R)**, Histograms showing the probability of confined tracks occurring along long (*x*) or short (*y*) axis of cells. Histograms correspond to panels **(G–L)**.

As further test for the idea that the MS2 tag with two repeats of the MS2-binding site is useful for SMT, we tracked molecule fluorescence under rifampicin stress. We used concentrations of rifampicin where not all RNAP molecules might be inhibited, so some transcription should remain active. Figure 6 and Supplementary Figure 4 show a similar change in the localization pattern of MS2-mVenus expressed without any binding site, and L1-mVenus, which showed exclusive accumulations at the cell poles (Figures 6A,B,E,F). mRNA *spoIIIE-ymfC* showed the strongest effect. Under normal conditions, *spoIIIE-ymfC* had a higher density at the cell periphery (Figure 6C), whereas following rifampicin treatment, membrane-proximal accumulation was lost; only some increased signal at the poles and the cell center remained (Figure 6D). With regard to the confinement maps, MS2-mVenus only and L1-mVenus changed their pattern by stronger polar accumulation, whereas *spoIIIE-ymfC* mRNA visually lost peripheral localization in favor of more central positioning (Figures 6G–L). Interestingly, histograms of confined tracks revealed stronger polar accumulation of MS2-mVenus only, L1-mVenus and *spoIIIE-ymfC* following RNA depletion, as judged from the *x*-axis scans (Figures 6M–R), whereas *y*-axis histograms reveal a shift of tracks away from the periphery of the cell toward the cell center, most pronounced for specifically labeled mRNA (Figures 6O,P). Strongest changes seen for *spoIIIE-ymfC* mRNA in response to mRNA depletion support the idea of significant specificity in mRNA labeling versus non-specific MS2-mVenus binding.

**FIGURE 7:**
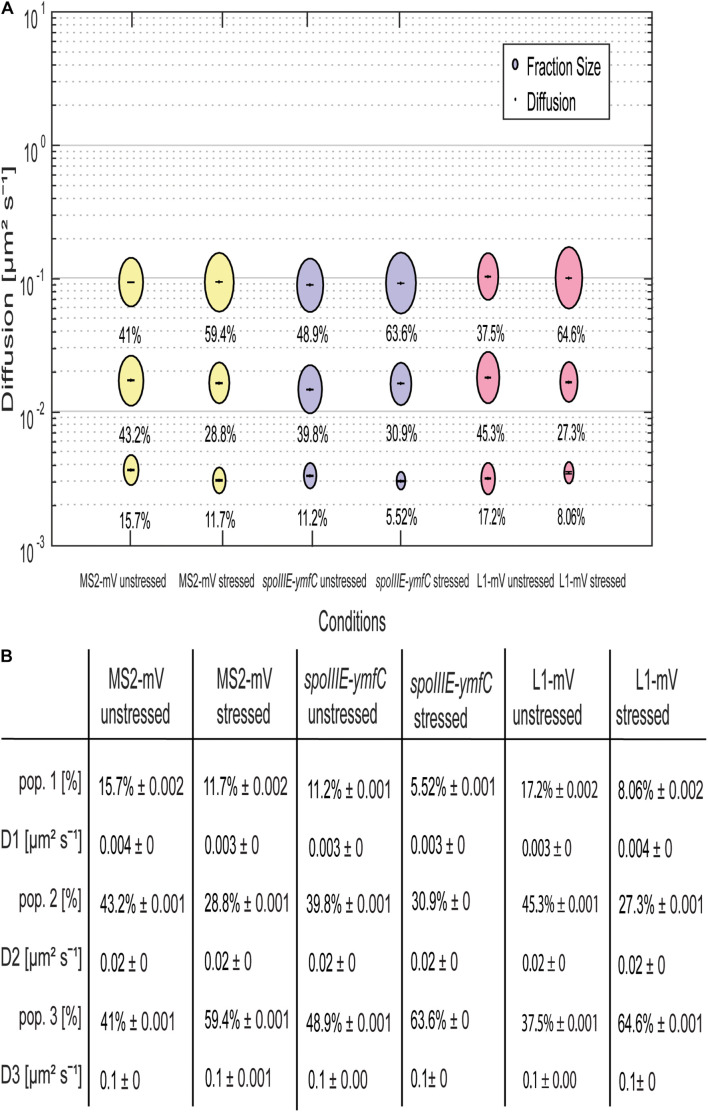
Rifampicin stress affects population sizes of mRNA and of ribosomal protein L1. For the determination of the diffusive coefficient and the fraction size, SQD was used. **(A)** The bubble plot shows the size of the fraction where each bubble is proportional to the area of its corresponding diffusion coefficients. MS2-mVenus is shown in yellow, whereas the mRNA operon *spoIIIE-ymfC* is shown in purple. L1-mVenus is colored in pink. In Table **(B)**—another way to display the SQD results—the data shown are the population sizes in% at a fixed DC that fits best to all three conditions, for better comparison if there are changes. For the stressed conditions, 50 μg/mL rifampicin was added to the cells for 40 min.

Not only the localization pattern changed following downshift of RNAP activity, but also the single-molecule dynamics (Figure 7). Here the effect was even more pronounced for *spoIIIE-ymfC* and L1 than for MS2 tag-only. *D*’s for the three populations remained similar for all three constructs (Figure 7B). Population size of the static fraction decreased for MS2-mVenus only; the intermediate mobile fraction also decreased under mRNA depletion, whereas the high-mobile population increased (Figure 7A). Thus, mRNA depletion led to higher mobility of MS2-mVenus. L1-mVenus showed a similar but stronger trend of increased mobility; the static fraction was less than halved, whereas the fastest (“free 50S subunits”) fraction almost doubled (Figure 7A). Interestingly, *spoIIIE-ymfC* dynamics changed differently from those of L1. The static mRNA population halved under rifampicin treatment, and the high-mobile population increased from 48.9 to 63.6%, whereas the intermediate-mobile population changed only little. This would fit to our idea that the static and intermediate mobile populations of L1 and the tested mRNAs are different stages of assembled ribosomes, in which the static population could be the translating ribosome, which are similarly affected by a reduction in transcription activity.

### Artificial mRNAs Behave Similar to Native mRNAs

**FIGURE 8:**
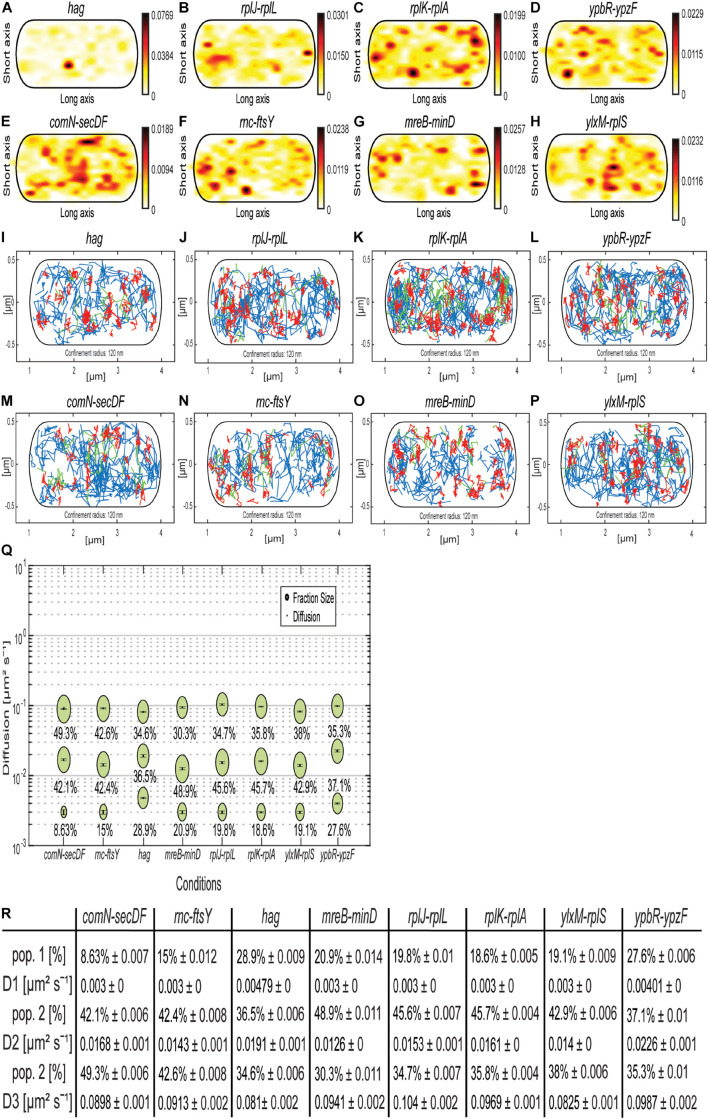
Artificially extended mRNAs approximately 1,700 bp longer than native mRNAs, with one MS2-binding site, behave similar to native mRNAs with two MS2-binding sites. **(A–H)** Heat maps. **(A–D)** are mRNAs for soluble proteins, and **(E–H)** for membrane-associated or membrane proteins. From white to red is the low to high probability of localization. **(A)** MS2-mVenus + *hag*_MS2–binding site 1 ×, **(B)** MS2-mVenus + *rplJ-rplL*_MS2–binding site 1 ×, **(C)** MS2-mVenus + *rplK-rplA*_MS2–binding site 1 ×, **(D)** MS2-mVenus + *ypbR-ypzF*_MS2–binding site 1 ×, **(E)** MS2-mVenus + *comN-secDF*_MS2–binding site 1 ×, **(F)** MS2-mVenus + *ftsY*_MS2–binding site 1 ×, **(G)** MS2-mVenus + *mreB-minD*_MS2–binding site 1 ×, and **(H)** MS2-mVenus + *ylxM-rplS*_MS2–binding site 1 ×. **(I–P)** Confinement maps. Blue freely diffusive tracks, red confined tracks (120-nm circle, minimum of eight steps), and green tracks with mixed behavior. **(I–L)** mRNAs for soluble proteins, **(M–P)** for membrane-associated or membrane proteins. **(I)** MS2-mVenus + *hag*_MS2–binding site 1 ×, **(J)** MS2-mVenus + *rplJ-rplL*_MS2–binding site 1 ×, **(K)** MS2-mVenus + *rplK-rplA*_MS2–binding site 1 ×, **(L)** MS2-mVenus + *ypbR-ypzF*_MS2–binding site 1 ×, **(M)** MS2-mVenus + *comN-secDF*_MS2–binding site 1 ×, **(N)** MS2-mVenus + *ftsY*_MS2–binding site 1 ×, **(O)** MS2-mVenus + *mreB-minD*_MS2–binding site 1 ×, and **(P)** MS2-mVenus + *ylxM-rplS*_MS2–binding site 1 ×. **(Q)** Bubble plots showing fraction size and diffusion constants of tested mRNAs. **(R)** Corresponding table showing population size and diffusion constants.

As shown, the MS2 tag apparently binds to an unspecific target in *B. subtilis.* Because of a fortuitous mistake in our initial design, plasmids containing mRNA and (initially a single) MS2 site did not contain a terminator sequence shortly after the MS2-binding site, like for the shown samples previously. A downstream terminator sequence in the plasmid, approximately 1,700 bp after the MS2-binding site, terminated the artificial mRNA constructs. We took advantage of this collection of artificial mRNAs (original mRNA at 5′ end, MS2 sequence in the middle, plasmid-derived 1,700 bp including terminator at the 3′ end) and observed the behavior of mRNAs having different 5′ portions. All constructs grew similarly as MS2-mVenus only–expressing cells (Supplementary Figure 2), suggesting that the extended mRNAs did not lead to any major problems for the cells. Even with one MS2 dimer bound to the MS2-binding site, we were able to track mRNA constructs (for JD analyses, see Supplementary Figure 5), whose static fractions were all considerably larger than for the cells expressing MS2-mVenus only (Figure 8Q), again suggesting that we receive enough specific labeling of tagged mRNAs. Regarding the mRNAs encoding for mostly soluble proteins (Figures 8A–D and Table 1), we see the highest density in the heat map near the membrane and also a small amount at the pole, whereas confined localization of the mRNAs of ribosomal proteins *rplJ-rplL* and *rplK-rplA* can be detected in the cytoplasm, probably around the nucleoid (Sohmen et al., 2015). The mRNA *hag*, which is monocistronic and encodes for flagellin, the largest extracellular part of the flagellum, is an exception (Holscher et al., 2018). Its mRNA is most evenly distributed and shows a strong midcell accumulation (Figure 8A and Supplementary Figure 6A), whereas the other mRNAs showed different variations of the peripherally and polarly accumulation theme (Figures 8B–K and Supplementary Figure 6). Interestingly, the heat maps for the two *ypbR-ypzF* constructs with two MS2 sites (Figures 5A,E) and one site and artificial 3′ end (Figures 8D,L) are visually similar to each other, and likewise for mRNAs *rnc-ftsY* and the operon *mreB-minD* (Figures 5, 8). This finding suggests that our observations made with the artificial mRNAs bear close resemblance to the ones made with the native constructs including only the two MS2-binding sites.

ComN is a transcription factor localized at the cell poles, whose transcript has also been shown to localize to the poles using FISH (dos Santos et al., 2012), and SecDF are integral membrane proteins involved in protein membrane insertion (Furukawa et al., 2017; Neef et al., 2020). *ylxM-rplS* encodes for SRP and ribosomal proteins, but also a tRNA methyltransferase (Bystrom and Bjork, 1982; Sohmen et al., 2015; RNase for 16S rRNA processing – rimM gene, Lovgren et al., 2004). Thus, upper panels of the confinement maps from Figure 8 show mRNAs for operons containing ORFs for soluble proteins (or membrane-associated DynA for the *ypbR* operon), and lower panels represent mRNAs encoding membrane proteins (or membrane-associated FtsY, Table 1). For all mRNAs, freely diffusive tracks are found throughout the cells, that is, also including movement through the nucleoid-containing cellular spaces. Although histograms of confined tracks show visual differences in membrane enrichment (depletion from the cell center), as judged from the *y*-axis scans, overall, there is no convincing general trend for mRNAs containing ORFs for membrane proteins toward the cell membrane (Supplementary Figures 6E–H), compared to those encoding soluble proteins (Figures 6A–D); a majority of mRNAs tend to be depleted from the central cellular area (Supplementary Figure 6).

Concerning diffusion constants, for both *rnc-ftsY* constructs, these are comparable among all three populations, but the population size of the mobile population for two binding sites with 42.6% is approximately 50% larger than of *rnc-ftsY* with one binding site (27.8%), and the static population shrinks by 50% from 30.2 to 15%. This change in dynamics could reflect lowered translation efficiency of the artificially enlarged mRNA. For *mreB-minD*, no significant changes in the population sizes were observed, but DCs differ. Interestingly, with one binding site, D_1_ (static) and D_2_ (slow mobile) are less mobile than with two repeats. Despite several other differences between the constructs, the general concept of three diffusive states for mRNAs holds true.

The largest differences between D’s were found for the slow mobile populations, the lowest of 0.013 μm^2^ s^–1^ for *mreB/minD* and the highest of 0.023 μm^2^ s^–1^ for *ypbR-ypzF.* Monocistronic mRNA *hag*, possibly containing the lowest number of translating ribosomes (we assume), appears to diffuse almost fastest (Figures 8Q,R). D_3_ of the different mRNAs varied between 0.081 and 0.104 μm^2^ s^–1^ (*hag* with the lowest and *rplJ-rplL* with the highest DC). Thus, assuming that the fast-mobile fraction corresponds to freely moving mRNA, size variations cause minor changes in mobility.

## Discussion

Until recent developments of high and super resolution imaging methods, it was thought that transcription and translation in bacteria happen in a temporally and spatially coupled manner (Miller et al., 1970). However, it has been shown that in *B. subtilis* and in *E. coli*, ribosomes are largely excluded from the central nucleoids, and approximately only 4% overlap with RNAPs (Bakshi et al., 2012), which are found at the nucleoid (Chai et al., 2014). This leads to the assumption that transcription and translation are spatial and temporarily separated. This does not account for all bacterial species, because in *C. crescentus*, chromosome loci, corresponding mRNA and ribosomes were found in close proximity, such that mRNAs stay near their transcript site (Montero Llopis et al., 2010).

In this study, we set out to study mRNA dynamics and localization at the highest possible resolution. We chose *B. subtilis*, an established model organism for Gram-positive bacteria, where it is known that transcription and translation occur in a spatially separated manner (Lewis et al., 2000).

Using the MS2 system of the bacteriophage MS2 (Stockley et al., 1995) and SMT, we used only two MS2-binding sites integrated into the 3′ end of four mRNAs, in order to cause as little deviation from native mRNAs as possible. With the caveat that MS2 appears to bind to some (likely ribosomal) RNA species in *B. subtilis* cells in a non-specific manner, we show that it is in principle possible to follow the movement of tagged sites on mRNA in a milliseconds time scale, assuming that individually tagged sites move similar to the entire mRNA molecule. Even given the noise generated by non-specifically binding MS2-mVenus protein, we found evidence for mRNA movement through the entire cell and, for confined motion, indicative of mRNA being within a larger structure such as bound by translating ribosomes. In agreement with the finding that free ribosomal subunits can move throughout the cells, while translating 70S ribosomes and polysomes are present at polar regions and at peripheral site surrounding the nucleoids (Sanamrad et al., 2014; Mayer et al., 2021), we found confined motion predominantly at sites excluded from the nucleoids, that is, the cell periphery and the cell poles. Although single-molecule data could be best explained by assuming three distinct populations of molecules with different average diffusion constants, we cannot exclude that only statistically positioned mRNAs (engaged in translation) and freely mobile molecule exist. Whether there are two or three populations does not change the major finding of considerably fast diffusion for mRNA molecules, be it in complex with ribosomes or as free mRNA molecules. Experiments using inhibition of transcription supported this view. Thus, we conclude that mRNA molecules are not generally excluded from the nucleoids but can diffuse through this subcellular space, allowing freedom for reaching different subcellular sites. Although we cannot prove that the fast-mobile fraction observed consists of MS2-mVenus specifically bound to the investigated mRNAs or to the non-specific RNA species, clearly the slow-mobile/static and medium-mobile fractions contain a large degree of specifically labeled mRNA molecules. Assuming a D of 0.02 μm^2^/s for medium-mobile diffusing mRNA molecules and taking this constant as an estimate for three-dimensional diffusion, on average, such a molecule would need less than 20 s to diffuse through a 3-μm-large (spherical) cell, using *t* = *r*^2^/6*D*. Assuming *D* of 0.01 for freely diffusing mRNAs, this number would drop to less than 4 s. Thus, mRNA molecules appear to have the possibility to reach different regions in a bacterial cell in a time frame of few seconds. Interestingly, diffusion of mRNA in eukaryotic cells has been determined to occur in a similar range (Katz et al., 2016; Yan et al., 2016), although proteins generally show higher diffusion constants in eukaryotic than in bacterial cells.

We found little evidence for the assumption that mRNAs encoding for membrane proteins are localized and translated at the cell membrane (Buxbaum et al., 2015). In general, all tested mRNAs, which consisted of operons or of monocistronic mRNAs, showed confined motion at sites surrounding the nucleoids, that is, close to the cell membrane. mRNA operons encoding for membrane and cytosolic proteins—such as *spoIIIE-ymfC* and *ylxM-rplS*—showed different degrees of membrane proximity for confined tracks but did not show clearly distinguishable patterns than mRNAs encoding solely soluble proteins, such as operons for ribosomal proteins. For mRNA encoding for flagellin that is secreted through the flagellar machinery to form the filament outside of cells, we found a marked localization to the cell middle. As a caveat to these experiments, confined tracks for MS2-mVenus only also showed a preference for polar localization. However, given that half or more confined tracks for specifically labeled mRNAs can be accounted for by specific binding of MS2-mVenus, a generally higher degree of membrane proximity for mRNAs encoding membrane proteins, and thus for translation of membrane proteins at the membrane, should have been visible, but histograms of confined tracks did not show such a clear trend, although different patterns between mRNAs were apparent.

Our data are not in conflict of studies reporting stronger association of mRNAs encoding for membrane proteins with membranes than for mRNAs containing ORFs for soluble proteins observed in *E. coli* (Benhalevy et al., 2015; Moffitt et al., 2016). It is also possible that although most mRNAs appear to be translated close to the cell membrane and at the cell poles, as seen in our study, mRNAs encoding membrane proteins are directly associated with the membrane, which is clearly the case of SRP-mediated membrane association of ribosomes translating nascent chains for membrane proteins.

Interestingly, observed diffusion constants for different mRNAs were rather similar. The populations with the highest DC, which we interpret to represent freely mobile molecules, showed a mobility much lower than that of even large proteins (Schibany et al., 2018; Mayer et al., 2021), in agreement with the large size of mRNAs. Interestingly, these molecules diffused even through the nucleoids that have been discussed to present diffusion barriers within bacterial cells. Based on an optimal fitting of data, we found two additional populations, one with an extremely low DC of essentially static motion, between 0.003 and 0.005 μm^2^ s^–1^, likely composed of mRNA covered with many ribosomes (polysomes). Inhibition of RNAP strongly decreased this population, supporting the idea of this population being involved in the translation process. An intermediate mobility fraction might be composed of mRNAs bound to 30S initiation complexes, that is, transition complexes; this population also decreased in size upon inhibition of translation.

To our knowledge, our study is the first report for DCs of ribosomal proteins in a Gram-positive bacterium. The observation of three diffusive populations agrees with findings made in *S. putrefaciens* (Mayer et al., 2021). A single DC was determined for freely diffusive ribosomal subunits or for translating ribosomes in *E. coli* (Sanamrad et al., 2014). Although our data can also be explained assuming two populations, we suggest that transition states between free subunits and translating 70S ribosomes/polysomes are reflected by out-tracking analyses, because we implemented criteria to avoid overfitting of data (Bayesian information criterion) and implemented several statistical tests to find the optimal fits for observed data. Dynamics determined for mRNAs were similar to intermediate-mobile and static ribosome populations, supporting the idea of mRNAs diffusing freely, or more slowly as initiation complexes, and basically arresting at peripheral sites in the cell as translation complexes.

It has been argued that mRNAs can have specific sites of location within the non-compartmentalized bacterial cell (Nevo-Dinur et al., 2011; Fei and Sharma, 2018). Our data are in no way contradictory, as we did observe differences in the pattern of localization of confined molecules for different mRNAs. In addition, polar localization described for mRNAs (dos Santos et al., 2012) is in agreement with our observation that confined motion of mRNAs (i.e., translation complexes) is prevalent at the polar areas of the cell. Thus, although out study failed to generate a system that is entirely specific for the labeled mRNAs to be studied, but is heavily blurred by non-specific binding of MS2 to unknown targets in *B. subtilis* (likely ribosomal RNA), our study provides fundamental data on single-molecule dynamics of mRNA molecules and is thus an important step toward understanding molecule dynamics in bacteria in real time and in understanding the intriguing asymmetric distribution of RNA molecules (Moffitt et al., 2016; Kannaiah et al., 2019) in a non-compartmentalized cell.

## Materials and Methods

### Bacterial Strains and Growth Conditions

Each *B. subtilis* construct is in the wild-type NCIB 3610, which possesses a plasmid for increased transformation efficiency with a *comI* mutation (Konkol et al., 2013). For cloning, the *E. coli* strain DH5α was used. Both strains were cultivated in Luria–Bertani medium, for *B. subtilis* only for overnight cultures. *E. coli* was grown at 200 revolutions/min (rpm) and 37°C, whereas *B. subtilis* was cultivated at 250 rpm and 30°C. For SMT, *B. subtilis* was grown in S7_50_ minimal medium [1% (wt/vol) fructose, 0.1% (wt/vol) glutamate, 0.004% (wt/vol)] Casamino acids (Jaacks et al., 1989) with the same temperature and speed. For the determination of the growth rate, it was measured with an optical density at 600 nm (OD_600_). An OD of 0.7 was used for microscopy. Selection of the strains was accomplished by using the antibiotics ampicillin (100 μg/mL) for *E. coli* and chloramphenicol (5 μg/mL) and spectinomycin (100 μg/mL) for *B. subtilis*. For mRNA depletion, commonly 200 μg/mL is used, which also rapidly induces cell death in the culture. We used a lower concentration of 50 μg/mL, where dead cells were rarely seen during the first 40 min after drug addiction. An even lower concentration of 25 μg/mL was also used (Supplementary Figure 4).

### Strain Construction

First, the fusion protein MS2-mVenus was constructed in *E. coli* DH5α with the plasmid pSG1193 (ECE153). With this plasmid, the coat protein is set between the flanks of the alpha amylase *amyE*. After transformation in *B. subtilis*, a double crossover occurs with the *amyE* gene section between plasmid and bacterial chromosome, and therefore, this gene is interrupted and no longer functional in *B. subtilis.* With this plasmid, a xylose promoter is set before the MS2-mVenus and therefore can be induced with xylose, something that was not done here. The strain MS2-mVenus in *B. subtilis* NCIB 3610 was then later on used to get the different mRNA constructs transformed into it.

For the mRNA constructs, the plasmid pHJDS (Defeu Soufo and Graumann, 2004) with a C-terminal fusion of one or two MS2-binding sites was used. After having the monocistronic and polycistronic mRNA constructs cloned with the MS2-binding site in *E. coli*, the constructs were cloned via a single-crossover event into *B. subtilis* MS2-mVenus at the original locus. Selection for the correct constructs was done by antibiotic resistance selection and test PCR. L1-mVenus was made by using the plasmid pSG1164 with a C-terminal mVenus. With this plasmid, also a single-crossover event occurs at the original locus in *B. subtilis.*

### Preparation of NCIB 3610 Constructs for Microscopy

*Bacillus subtilis* NCIB 3610 cells with only MS2-mVenus, MS2-mVenus + mRNA constructs, and L1-mVenus constructs were grown in S7_50_ minimal medium at 30°C under shaking conditions to an OD of 0.7. For the cells, stressed with 25 and 50 μg/mL rifampicin, it was added to the constructs and incubated for 40 min. Cells were spotted on coverslips (25 mm, Marienfeld) and covered with an agarose pad [1% (wt/vol)], made of S7_50_ Medium and a smaller coverslip (12 mm, Marienfeld).

### Single-Molecule Tracking, Data Acquisition, and Analysis

Imaging was performed with a Nikon Eclipse Ti microscope equipped with a high numerical aperture objective (CFI Apochromat TIRF 100XC Oil, NA 1.49), an EM-CCD camera (ImagEM X2, Hamamatsu), and a YFP filter set (BrightLine 500/24, Beamsplitter 520 and BrightLine 542/27). mVenus fluorophores were excited by the central part of a laser beam (TOPTICA Beam Smart, 515 nm, maximum power 100 mW) with a laser intensity of 20 mW. Each movie consists of 3,000 frames and was recorded with an integration time of 8, 20, or 75 ms, using Nikon NIS-Elements BR.

First, the videos were cut with Fiji (ImageJ) (Schindelin et al., 2012), and the first 500 to 1000 frames were cut off, to a point where only one or two signals were present in cells. Afterward, the cell meshes were set with oufti (Paintdakhi et al., 2016). For particle detection, U-track (Jaqaman et al., 2008), a MATLAB software, was used. Here, the minimal length of tracks was set to 8, to avoid analyzing freely diffusive molecules that diffuse slowly for a short time, and to link to points, no gaps for the particle detection was allowed. The Brownian search radius was set with the lower bound of 0 and the upper bound of 3. Data were analyzed using SMTracker (Rösch et al., 2018). Here, the Stationary Localization Analysis panel for the dwell time analysis and the SQD panel were used. *R*^2^ was as a measure for goodness, and statistical tests such as Kolmogorov–Smirnov goodness of fit and null hypothesis significance were used to find the best fit to Rayleigh distributions. Bayesian information criterion was used to avoid overfitting of data. Although, at present, SMTracker allows only to differentiate up to 3 populations, assuming more than 3 population presented overfitting of data from this study, based on an *R* value of “1” for using three populations.

With regard to the number of tracks (Supplementary Table 1), the MS2 system with two binding sites might be a better option to collect data for mRNA dynamics; however, the dynamics and localization patterns were quite similar between the one and two MS2-binding sites constructs. Thus, motion of artificial mRNAs does not appear to differ much between native and synthetic mRNAs.

## Data Availability Statement

The original contributions presented in the study are included in the article/Supplementary Material, further inquiries can be directed to the corresponding author/s.

## Author Contributions

LS performed all the experiments, analyzed the data, and helped to write the manuscript. PG devised of the study, supervised all the experiments, helped to analyze the data, and wrote the manuscript. Both authors contributed to the article and approved the submitted version.

## Conflict of Interest

The authors declare that the research was conducted in the absence of any commercial or financial relationships that could be construed as a potential conflict of interest.

## Publisher’s Note

All claims expressed in this article are solely those of the authors and do not necessarily represent those of their affiliated organizations, or those of the publisher, the editors and the reviewers. Any product that may be evaluated in this article, or claim that may be made by its manufacturer, is not guaranteed or endorsed by the publisher.
