# Supplementary material for: Real-Time Messenger RNA Dynamics in Bacillus subtilis
Source: Front Microbiol. 2021 Nov 18;12:760857. doi: 10.3389/fmicb.2021.760857 (PMC8637298; doi:10.3389/fmicb.2021.760857)
Supplement: Supplementary file 1 [file Data_Sheet_1.pdf]

Supplemental data

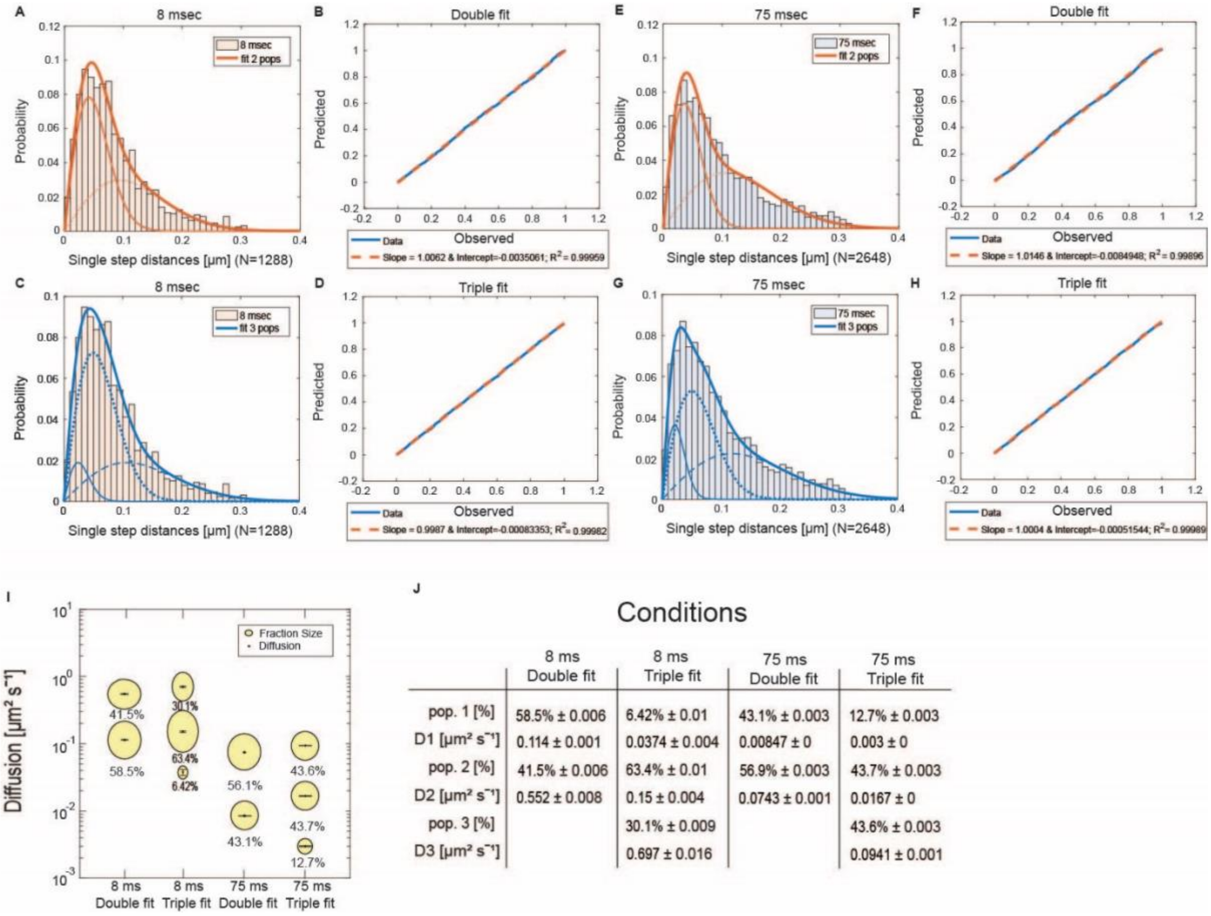

	8 ms Double fit	8 ms Triple fit	75 ms Double fit	75 ms Triple fit
pop. 1 [%]	58.5% ± 0.006	6.42% ± 0.01	43.1% ± 0.003	12.7% ± 0.003
D1 [μm² s⁻¹]	0.114 ± 0.001	0.0374 ± 0.004	0.00847 ± 0	0.003 ± 0
pop. 2 [%]	41.5% ± 0.006	63.4% ± 0.01	56.9% ± 0.003	43.7% ± 0.003
D2 [μm² s⁻¹]	0.552 ± 0.008	0.15 ± 0.004	0.0743 ± 0.001	0.0167 ± 0
pop. 3 [%]		30.1% ± 0.009		43.6% ± 0.003
D3 [μm² s⁻¹]		0.697 ± 0.016		0.0941 ± 0.001

Fig. S1 A minimum of two populations for MS2 protein are shown by the jump distance analysis. JD analysis shows the distribution of the particles' displacements in a fixed time interval, plotted in a histogram (A,C,E,G). The probability-probability plots display the goodness of the fit of predicted (red dotted lines) and measured data, shown with the blue solid lines (B,D,F,H). All triple fit models are shown in blue (C,G), double fits are depicted in red (A,E). Different dotted lines represent the subpopulations for a double or triple fit, while the solid lines represent the totality of the subpopulations. Double and triple fit, as well as the quantile-quantile plot in comparison to each other for the two different exposure times of the tracked MS2-mVenus fusion. (A) shows the double fit for the MS2 tag tracked with 8 ms exposure time and its belonging quantile-quantile plot (B). 75 ms is the other tested exposure time, with the double fit (E) and quantile-quantile plot (F). The triple fit for MS2-mVenus tracked with an exposure time of 8 ms (C), its quantile-quantile plot (D) and tracked with 75 ms exposure time (G) and its belonging quantile-quantile plot (H) are shown. For the determination of the diffusive coefficient and the fraction size, square displacement analysis (SQD) was used (I). The bubble plot shows the size of the fraction where each bubble is proportional to the area of its corresponding diffusion coefficients. Table (J) –displays SQD results - the shown data are the population sizes in % at its fixed, corresponding diffusion coefficient [μm^{2}s^{-1}] for each condition.

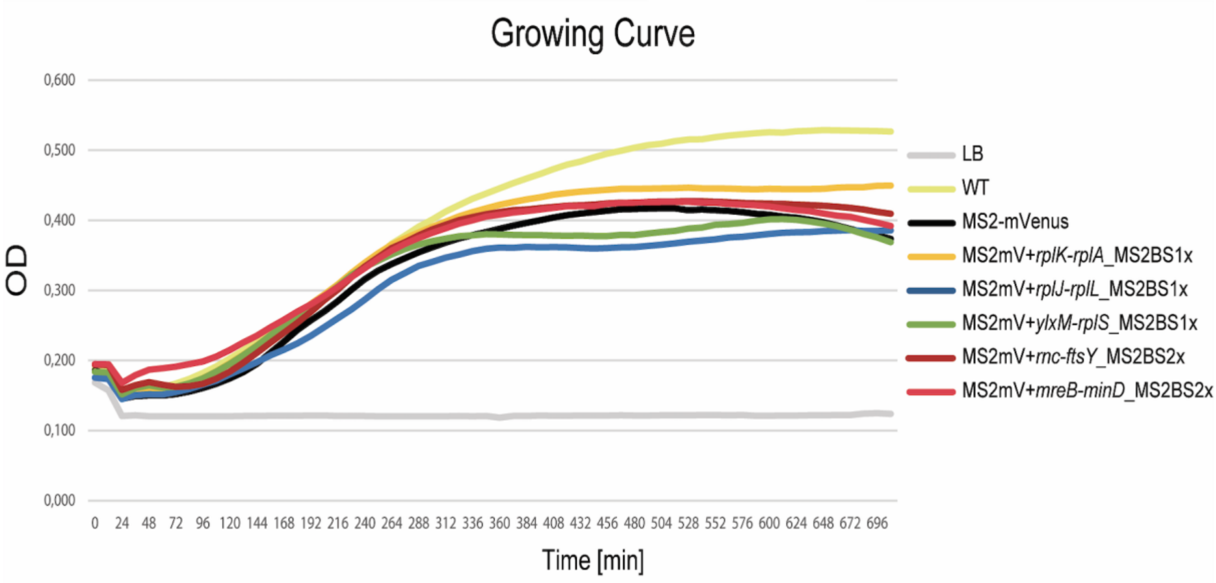

Fig. S2 Growth curves of constructs with the MS2-mVenus fusions. Every 12 minutes, measurement of the optical density (OD) was done. For each condition, cells were grown in a 96 well plate with the rich media Luria-Bertani (LB). Each condition consists of a biological triplicate, done on three different days. Each replicate consists of eight technical replicates. The LB condition is growth media without cells, that acts as a control for the OD (in grey). WT is the other control for the growing behavior of *B. subtilis*, which consists of the *B. subtilis* wild type 3610 without any fusion, shown in yellow. MS2-mVenus is the MS2 coat protein with a mVenus fusion, shown in black. Every mRNA construct consists also of the MS2-mVenus fusion. With one MS2 binding sites are the mRNAs MS2-mVenus +*rplK-rplA*_MS2BS1x (orange), MS2-mVenus + *rplJ-rplL*_MS2BS1x (blue) and MS2-mVenus + *ylxM-rplS*_MS2BS1x (green). With two MS2 binding sites are the mRNA constructs MS2-mVenus + *ftsY*_MS2BS2x (dark red) and MS2-mVenus + *mreB-minD*_MS2BS2x (red).

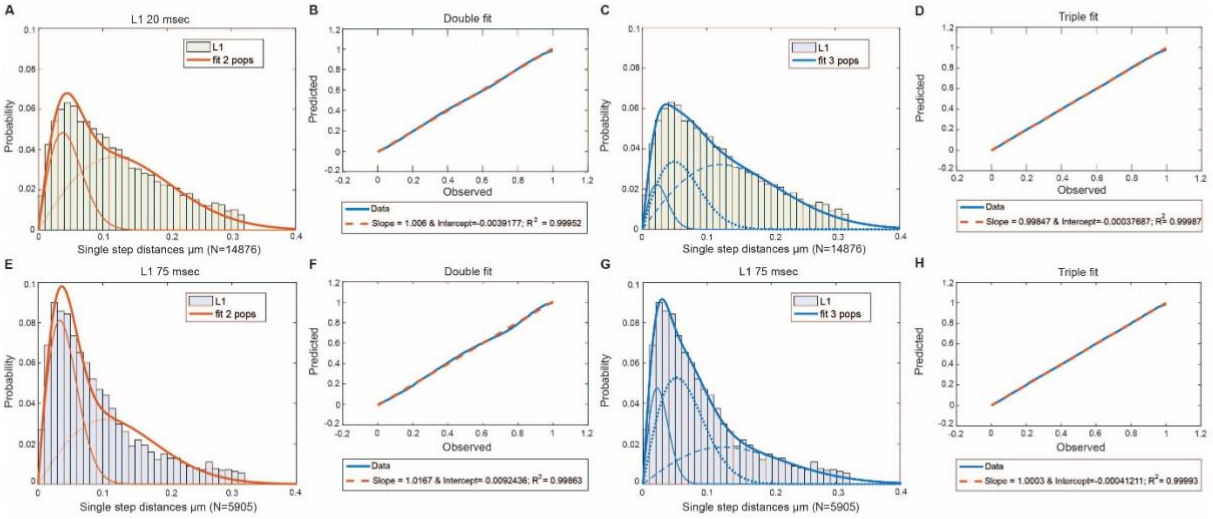

Fig. S3 Jump distance analysis of the tracked ribosomal protein L1 with two different exposure times. Jump distance analysis shows the distribution of the particles' displacements in a fixed time interval, plotted in a histogram (A,C,E,G). The probability-probability plot displays the goodness of the fit of what is predicted (red dotted lines) and how the data actually behaves, shown with the blue solid lines (B,D,F,H). All triple fit models are shown in blue (C,G), double fits are depicted in red (A,E). Different dotted lines represent the subpopulations for a double or triple fit, while the solid lines represent the totality of the subpopulations. Double and triple fit, as well as the quantile-quantile plot in comparison to each other for the three different exposure times of the tracked L1. (A) shows L1 tracked with 20 ms exposure time and its belonging quantile-quantile plot (B). For the tracking condition with 75 ms, the double fit (E) and the quantile-quantile plot (F) are shown. The triple fit for L1 tracked with an exposure time of 20 ms (C), its quantile-quantile plot (D), and tracked with 75 ms exposure time (G) and its quantile-quantile plot (H) are shown.

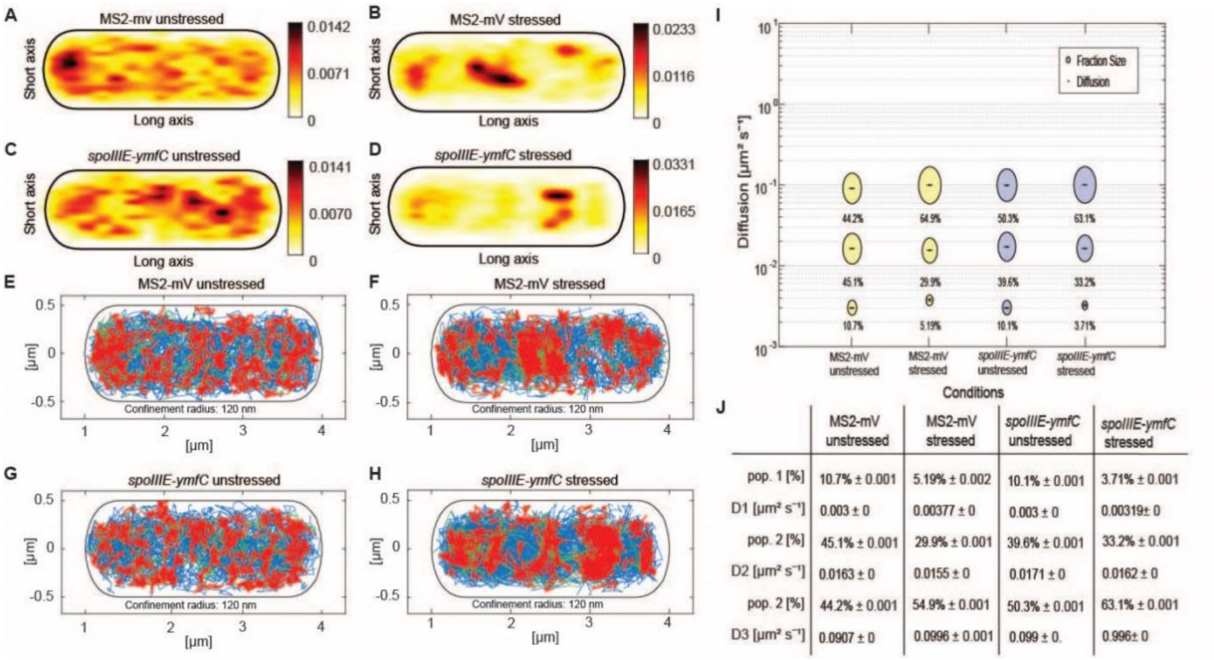

	MS2-mV unstressed	MS2-mV stressed	*spoIIIE-ymfC* unstressed	*spoIIIE-ymfC* stressed
pop. 1 [%]	10.7% ± 0.001	5.19% ± 0.002	10.1% ± 0.001	3.71% ± 0.001
D1 [µm² s⁻¹]	0.003 ± 0	0.00377 ± 0	0.003 ± 0	0.00319± 0
pop. 2 [%]	45.1% ± 0.001	29.9% ± 0.001	39.6% ± 0.001	33.2% ± 0.001
D2 [µm² s⁻¹]	0.0163 ± 0	0.0155 ± 0	0.0171 ± 0	0.0162 ± 0
pop. 2 [%]	44.2% ± 0.001	54.9% ± 0.001	50.3% ± 0.001	63.1% ± 0.001
D3 [µm² s⁻¹]	0.0907 ± 0	0.0996 ± 0.001	0.099 ± 0.	0.996± 0

Fig. S4 Analyze of the coat protein and the mRNA *spoIIIE-ymfC* under Rifampicin stress. For the stressed conditions, 25 µg/ml Rifampicin were added to the cells for 40 minutes. (A-D) In standardized cells of 1 x 3 µm, all tracks of the different fusions are projected. From white to red is the low to the high probability of distribution and spatial localization of the tracks represented for tracking with an exposure time of 75 ms for the (A) unstressed and (B) stressed MS2-mVenus. (C) is the unstressed and (D) stressed mRNA MS2-mVenus + *spoIIIE-ymfC*_MS2 binding site 2x. (E-H) Also in standardized cells of 1 x 3 µm, all tracks of the two constructs are depicted. Blue represents free diffusive tracks, red are tracks that are restricted to a confined movement in a 120 nm circle with a minimum of 8 steps. In green are shown tracks with a mixed behavior between mobile and confined movement and vice versa. (E) is the MS2 tag MS2-mVenus without stress and in (F) under Rifampicin stress. (G) is the unstressed MS2-mVenus + *spoIIIE-ymfC*_MS2 binding site 2x and (H) the condition of the mRNA under Rifampicin stress. For the determination of the diffusive coefficient and the fraction size, square displacement analysis (SQD) was used (I). The bubble plot shows the size of the fraction where each bubble is proportional to the area of its corresponding diffusion coefficients. It can be distinguished between 3 populations, a static (lower bubbles), a slow mobile (middle bubbles) and a mobile (upper bubbles) fraction. In table (J) – another way to display the SQD results - the shown data are the population sizes in % at its fixed, corresponding diffusion coefficient [$\mu m^2 s^{-1}$] for each condition.

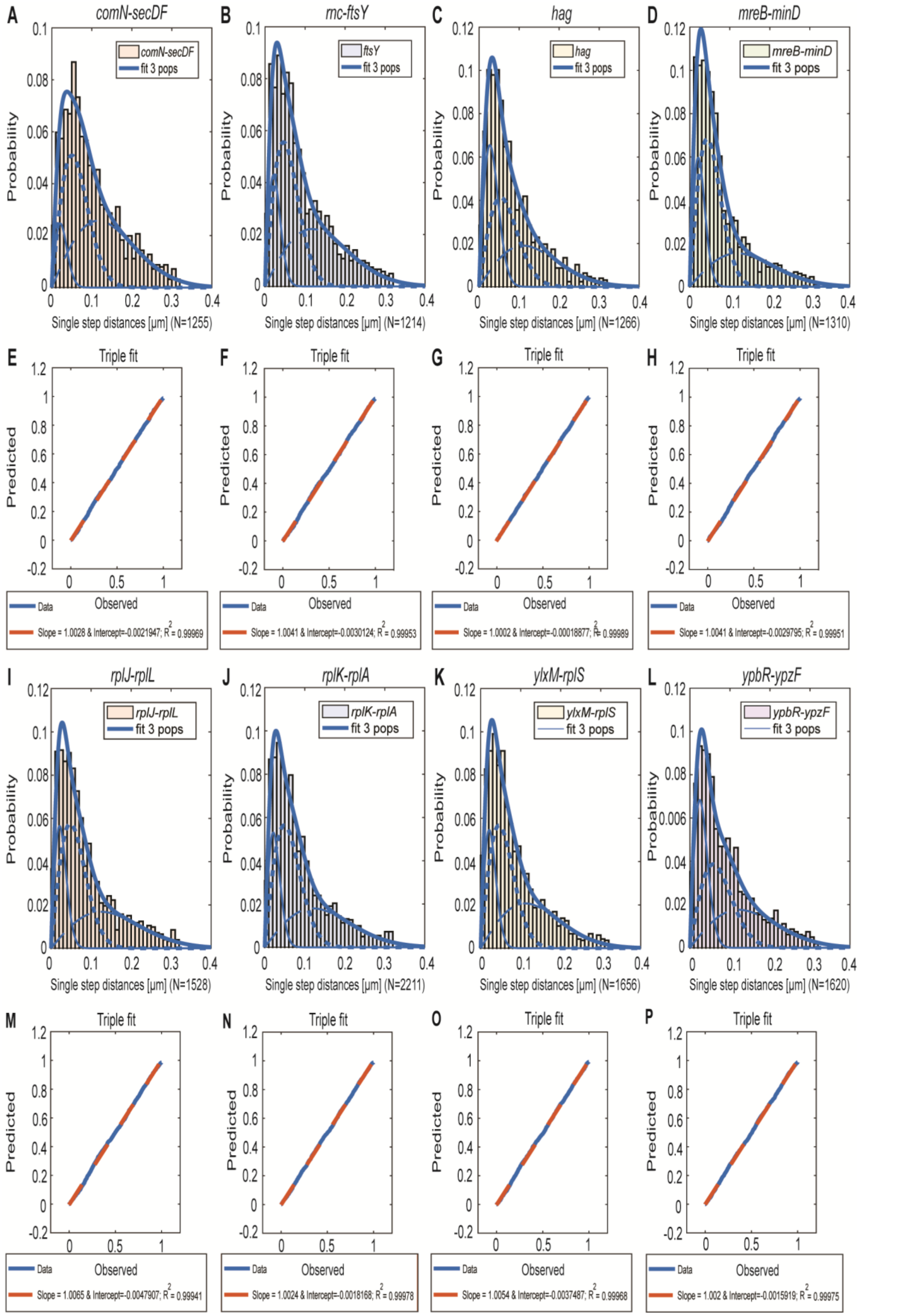

Fig. S5 Triple fit of eight different artificial mRNAs with one MS2 binding sites analyzed with the jump distance analysis. JDA shows the distribution of the particles' displacements in a fixed time interval, plotted in a histogram (A-D,I-L). The probability-probability plot displays the

goodness of the fit of what is predicted (red dotted lines) and how the data actually behaves, shown with the blue solid lines (E-H,M-P). All triple fit models are shown in blue. Different dotted lines represent the subpopulations for a triple fit, while the solid lines represent the totality of the subpopulations. The triple fit model of the jump distance analysis was chosen for all mRNAs: (A) MS2-mVenus + *comN-secDF*_MS2 binding site 1x and the belonging quantile-quantile plot (E), (B) MS2-mVenus + *ftsY*_MS2 binding site 1x and the belonging quantile-quantile plot (F), (C) MS2-mVenus + *hag*_MS2 binding site 1x and the belonging quantile-quantile plot (G), (D) MS2-mVenus + *mreB-minD*_MS2 binding site 1x and the belonging quantile-quantile plot (H), (I) MS2-mVenus + *rplJ-rplL*_MS2 binding site 1x and the belonging quantile-quantile plot (M), (J) MS2-mVenus + *rplK-rplA*_MS2 binding site 1x and the belonging quantile-quantile plot (N), (K) MS2-mVenus + *ylxM-rplS*_MS2 binding site 1x and the belonging quantile-quantile plot (O) and (L) MS2-mVenus + *ypbR-ypzF*_MS2 binding site 1x with its belonging quantile-quantile plot (P).

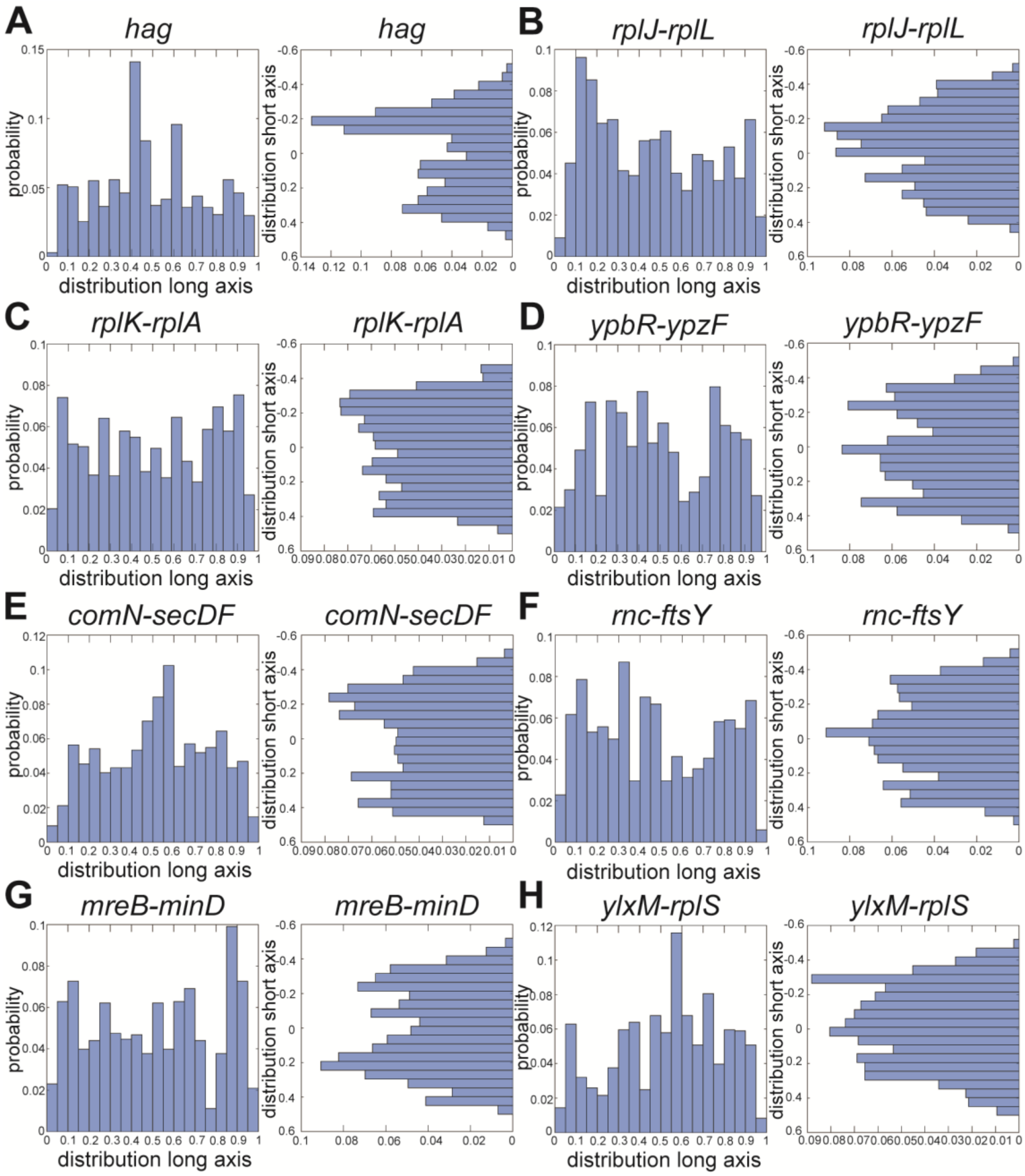

Fig. S6 Histograms of the probability of confined tracks along long (x) – or short (y) axis of cells. Histograms correspond to Fig. 8 I-P.

Table S1

A

	ypbR-ypzF	*ftsY*	*mreB-minD*	*spoIIIE-ymfC*
Cells	87	123	85	93
Cells with tracks	79	109	77	78
Tracks	297	651	245	263
Tracks per cell	4.0792	5.4100	3.2806	3.5907

B

	hag	*rplJ-rplL*	*rplK-rplA*	*ypbR-ypzF*	*comN-secDF*	*ftsY*	*mreB-minD*	*ymfC_rplS*
Cells	71	56	94	80	90	62	62	118
Cells with tracks	52	44	64	63	55	42	52	74
Tracks	110	136	190	150	113	113	123	158
Tracks per cell	2.1159	3.0571	2.4958	2.6179	1.9354	2.6333	2.3835	1.9970

(A) shows the statistics for the mRNAs with two MS2 binding sites, (B) are the artificial mRNAs with one MS2 binding site.
